# New Insights into the Education of Children with Congenital Heart Disease with and without Trisomy 21

**DOI:** 10.3390/medicina59112001

**Published:** 2023-11-14

**Authors:** Katharina R. L. Schmitt, Laura K. Sievers, Alina Hütter, Hashim Abdul-Khaliq, Martin Poryo, Felix Berger, Ulrike M. M. Bauer, Paul C. Helm, Constanze Pfitzer

**Affiliations:** 1 Department of Congenital Heart Disease—Pediatric Cardiology, Deutsches Herzzentrum der Charité, Augustenburger Platz 1, 13353 Berlin, Germany; katharina.schmitt@dhzc-charite.de (K.R.L.S.); felix.berger@dhzc-charite.de (F.B.); 2 Charité—Universitätsmedizin Berlin, Corporate Member of Freie Universität Berlin and Humboldt-Universität zu Berlin, Charitéplatz 1, 10117 Berlin, Germany; 3 Competence Network for Congenital Heart Defects, 13353 Berlin, Germany; ulrike.bauer@dhzc-charite.de; 4 Department of Internal Medicine I, Christian-Albrechts-University and University Hospital Schleswig-Holstein, Campus Kiel, 24105 Kiel, Germany; l.k.sievers@ikmb.uni-kiel.de; 5 Department of Pediatric Cardiology, Saarland University Medical Center, 66421 Homburg, Germany; hashim.abdul-khaliq@uks.eu (H.A.-K.); martin.poryo@uks.eu (M.P.); 6 National Register for Congenital Heart Defects, 13353 Berlin, Germany

**Keywords:** congenital heart disease, trisomy 21, neurodevelopment, education, school career

## Abstract

*Background and Objectives*: Patients with congenital heart disease (CHD), especially as a concomitant syndromal disease of trisomy 21 (T21), are at risk for impaired neurodevelopment. This can also affect these patients’ education. However, there continues to be a research gap in the educational development of CHD patients and T21 CHD patients. *Materials and Methods*: In total, data from 2873 patients from the German National Register for Congenital Heart Defects were analyzed. The data are based on two online education surveys conducted among patients registered in the National Register for Congenital Heart Defects (2017, 2020). *Results*: Of 2873 patients included (mean age: 14.1 ± 4.7 years, 50.5% female), 109 (3.8%) were identified with T21 (mean age: 12.9 ± 4.4 years, 49.5% female). T21 CHD participants had a high demand for early specific interventions (overall cohort 49.1%; T21 cohort 100%). T21 CHD children more frequently attended special schools and, compared to non-trisomy 21 (nT21) CHD patients, the probability of attending a grammar school was reduced. In total, 87.1% of nT21 CHD patients but 11% of T21 CHD patients were enrolled in a regular elementary school, and 12.8% of T21 CHD patients could transfer to a secondary school in contrast to 35.5% of nT21 CHD patients. Most of the T21 CHD patients were diagnosed with psychiatric disorders, e.g., learning, emotional, or behavioral disorders (T21 CHD patients: 82.6%; nT21 CHD patients: 31.4%; *p* < 0.001). *Conclusions*: CHD patients are at risk for impaired academic development, and the presence of T21 is an aggravating factor. Routine follow-up examinations should be established to identify developmental deficits and to provide targeted interventions.

## 1. Introduction

Congenital heart defects (CHD) are the most common organ malformation in newborns and the cause can be described as epigenetic [1]. Worldwide, CHD occur at a prevalence of approximately 1% and thus are significantly contributing to infant mortality and morbidity [2]. In recent decades, major improvements in the early diagnostics and surgical and interventional therapeutic approaches have led to significantly improved mortality and morbidities in these children [3,4,5,6]. Therefore, increased research interest has been focusing on the long-term outcome and residual sequelae in recent years [7]. Earlier studies have shown that patients with CHD are at risk for impaired neurodevelopment with deficits in motor function, cognition, and language [8,9,10,11]. These deficits may also affect areas of memory and executive function, visual–spatial imagination, attention, and social skills [12]. Thereby, the developmental opportunities of patients with CHD in terms of educational pathway and achieving a degree may be impaired. This can have far-reaching consequences for patients and their families: poorer chances for a high level of professional education and achievement in working life, and an absence of social integration and an independent life, which can all result in impairments in quality of life [13,14]. There is consensus that the presence of a genetic syndrome and the severity of the CHD are important influencing factors [15,16]. Trisomy 21 (T21) is the most common genetic cause of intellectual impairment and learning difficulties, and about 45 percent of children born with T21 have a CHD [17,18]. However, there are few and conflicting data on the scholastic development of patients with CHD, especially in this specific group of T21 CHD patients. Valid knowledge on their scholastic development would be very valuable for the scientific community, patients with CHD, and their parents. It is the prerequisite to identifying patients at risk and to enable early diagnostics and therapy.

Our study aims to describe the development of children with CHD with and without T21 in the school environment and to identify risk groups.

## 2. Materials and Methods

### 2.1. Study Design and Patient Cohort

In total, data from 2873 patients from the German National Register for Congenital Heart Defects could be included in the analysis. The data basis is formed by two online surveys on the topic of school and education, which were conducted among patients of the National Register for Congenital Heart Defects. Data collection took place in 2017 and 2020. In addition, the statistical analyses performed also included medical data from the National Register for Congenital Heart Defects medical database, which were updated/added/corrected as far as it was possible/required. In individual cases, the National Register for Congenital Heart Defects medical data may have been taken from periods prior to the patient survey if, for example, the National Register for Congenital Heart Defects did not have a more recent physician’s note. The medical data and the collected survey data were thoroughly checked for correctness, completeness, and plausibility, and only patients were included in whom the educational level of both parents was known and the severity of the CHD could be classified with medical certainty.

As part of the data protection concept of the National Register for Congenital Heart Defects registered with the Berlin Commissioner for Data Protection and Freedom of Information, no. 531.390, a data infrastructure has been established in the National Register for Congenital Heart Defects that enables the storage of (medical) data. General approval was obtained from the Ethics Committee of Charité—Universitätsmedizin Berlin, Germany, for all research conducted on the National Register for Congenital Heart Defects. In addition, the Ethics Committee of Charité—Universitätsmedizin Berlin, Germany, specifically approved both studies (No. EA2/137/17; EA2/190/19).

Cardiac diagnoses were assigned according to the International Pediatric and Congenital Cardiac Code (IPCCC) classification [19]. Following Warnes et al., CHD were classified into three groups: simple CHD, moderate CHD, and complex CHD. Furthermore, T21 CHD patients were identified according to the IPCCC. Parental education level was categorized as high, medium, or low according to the International Standard Classification of Education (ISCED).

### 2.2. Data Basis and Baseline Studies in Detail

STUDY I: The aim of the first study from 2017 was to assess the educational career of young and adolescent CHD patients [20]. To this end, an exploratory online survey was conducted among patients registered with the National Register for Congenital Heart Defects in Germany. This was a cross-sectional study. Accordingly, associations but not causalities could be investigated. Patients born between 1992 and 2011 were included in the study. For this purpose, the NRCHD medical database was searched for living patients from the defined birth period. These patients first had to be alive, second had to have agreed to participate in studies, and third had to have a current e-mail or postal address. Eligible patients were then invited to participate in the study. A total of 2609 CHD patients participated (female = 1870; 71.7%). The factors examined included, for example, age at enrollment, type of school, potential class repetition, and school graduation. The vast majority (83.4%) attended a regular elementary school. Most patients (73.3%) were enrolled at age 6 years or younger and 45.7% graduated from school with the qualification required for university study. Patients with mild CHD (57.3%), moderate CHD (47.5%), and complex CHD (35.1%) achieved a high school diploma [20]. Here, it is clear that patients with complex CHD are less likely to achieve a high school diploma. Overall, the results of this study show that, in general, a normal school career is possible for all CHD patients. However, the severity of CHD plays an important role and should be considered in school career planning.

STUDY II: In this study, the school career of CHD patients was investigated in 2020 [21]. Particular attention was paid to the consideration of head circumference. Accordingly, the aim was to compare CHD patients with normal head circumference with CHD patients with microcephaly in order to evaluate the influence of insufficient head circumference in CHD patients on school career. The cohort of patients studied was originally from a previous study of somatic development in children with CHD in 2018 (*n* = 2818) [22]. Somatic development is impaired in children with CHD and head circumference can be understood as a predictor of neurodevelopmental outcome. Accordingly, the aim was to determine current reference values for head circumference, body weight, and length/height. The study population of the 2818 young CHD patients consisted of patients from the PAN study (Prävalenz angeborener Herzfehler bei Neugeborenen in Deutschland) [23]. These patients were born in Germany between 2006 and 2009. Comparison with heart-healthy children revealed that in children with CHD all somatic measures had significantly lower values; this is particularly evident in children with severe CHD [22]. The results found indicate a concomitant brain pathology that does not appear to be related to possible cardiac surgery. Based on the PAN study [23] and the analysis of somatic parameters in 2018 [22], another online-based patient survey was then conducted in 2020 [21] among these 2818 patients to learn more about a possible impact of head circumference on school career. Patients were recruited through the National Register for Congenital Heart Defects medical database. For this purpose, living patients were identified from the 2818 patients. If they generally consented to study participation and a current e-mail or postal address was available, eligible patients were invited to participate in the study. A total of 750 (26.6%) of the 2818 patients participated in the online survey in 2020. Overall, 91 of the 750 patients (12.1%) were diagnosed with a CHD and microcephaly. The presence of microcephaly was found to be significantly associated with the severity of the CHD. In particular, developmental delays, disabilities, and learning or language disorders were significantly more common in microcephalic CHD patients [21]. Accordingly, 85.7% of microcephalic patients received early intervention (47.6% of non-microcephalic patients). There was no significant difference in school enrollment age (enrollment mostly at six years of age). However, only 51.6% of the microcephalic patients were enrolled in a regular elementary school (89.9% of the non-microcephalic patients) and only 14.3% attended a high school [21]. Microcephaly in CHD patients consequently increases the risk for impaired school development, which requires targeted interventions to optimize developmental potential.

Present study: In both Study I and Study II, the questions were partially identical. Accordingly, only identical questions were included in the analyses. Patients who participated in both online surveys were then identified. This was achieved using a nine-digit login code assigned to patients for study participation by the National Register for Congenital Heart Defects. This is therefore a pseudonymized data collection and evaluation without direct personal reference. If, for example, a patient had participated in both online surveys, the data from Study II were used in order to always include the most current information. This ensured that no patient appeared twice in the data set. Thus, to be included in the current analyses, patients had to have participated in either Study I or Study II. In addition, it was decided to include only patients in the statistical analyses if there was a clear main cardiac diagnosis and thus a severity classification was possible. Since parental education level is known to have a relevant influence on children’s schooling, parental education level was asked about in both online surveys and it was decided that patients would only be included in the analyses when complete information on parental education level was available from both parents. The criteria described were checked before creating the final data set of the present study. All cases that did not meet the above conditions were excluded from the subsequent analyses. By combining the data from Study I and Study II, it was possible to include in the analyses all patients from whom up-to-date information on schooling was available in the National Register for Congenital Heart Defects. This maximization of the number of cases made it possible to analyze a total of 2873 patients with a confirmed CHD diagnosis in order to obtain results that were as reliable and robust as possible. Particular emphasis was placed on data quality and accuracy in the collection and integration of survey and medical data. The respective implementation of the two online surveys was closely accompanied by multidisciplinary research teams. The study participants had the opportunity to contact the National Register for Congenital Heart Defects in writing or by telephone at any time in case of queries or problems. The pseudonymized data collection prevents socially desirable response behavior. The medical data are thoroughly quality-assured at the National Register for Congenital Heart Defects by specially trained medical staff and specialists. As described, high demands were also placed on data quality and the completeness of central data such as CHD severity and parental education level. Finally, the final data set from Study I and Study II was checked by independent scientists before the analyses were carried out.

### 2.3. National Register for Congenital Heart Defects

The National Register for Congenital Heart Defects was founded in 2003 by the three German cardiac medical societies (DGPK, DGTHG, DGK) as a non-profit scientific association and is currently funded by the German Federal Ministry of Education and Research (BMBF). The NRCHD functions as a core project in the Competence Network for Congenital Heart Defects, in which hospitals, heart centers, physicians in private practice, patient organizations, universities and research institutions, as well as, in sub-projects, statutory health insurance companies, cooperate. The National Register for Congenital Heart Defects is closely networked with all pediatric cardiology departments and pediatric cardiologists in private practice throughout Germany. The National Register for Congenital Heart Defects currently has around 60,000 patients registered with various CHD of all severities (as of October 2023) and is thus the largest CHD registry in Europe. The multicenter research approach makes Germany-wide representative online-based data collection and analysis on various topics possible [24]. The National Register for Congenital Heart Defects already has many years of experience with online surveys. The main focus is on project communication and coordination and a patient-centered research approach, which is ensured in each case by the established infrastructure of the National Register for Congenital Heart Defects.

### 2.4. The German School System

In Germany, school attendance is compulsory until the age of 15 years and is free of charge. Usually, children start elementary school at 5–7 years of age, but mainly at the age of 6 years. If a child has special needs in her/his educational, developmental, and learning possibilities (e.g., due to a learning or mental/cognitive disability or a sensory and/or physical disability, less frequently due to a long-term illness), she/he is introduced to a special school. After finishing primary education (4–6 years, depending on the federal state), there are several options for secondary schooling according to the student’s abilities: the highest is the grammar school (‘Gymnasium’), where pupils graduate after 8–9 years with a high school diploma, enabling them to study at university. Graduation from secondary school (usually after 9–11 years) allows students to start an apprenticeship [25].

### 2.5. Statistical Analyses

The statistical analyses performed are primarily descriptive. For group comparisons, the chi-square test was used for nominal/ordinal variables and the *t*-test for metric variables. Analyses were performed using the statistical software SPSS (IBM Corp. Released 2013. IBM SPSS Statistics for Windows, Version 22.0. Armonk, NY, USA: IBM Corp.).

## 3. Results

### 3.1. Patient Characteristics

Of the 2873 patients included, 50.5% were female. The average age at the time of data collection was 14.1 ± 4.7 years. CHD severity was as follows: 32.1% had a simple CHD, 36.9% had a moderate CHD, and 31% had a severe CHD (see Table 1). Ventricular septal defect (24.9%) and atrial septal defect (10.9%) were the most frequent CHD in the analyzed patient cohort. Table 2 shows the most frequent CHD phenotypes in detail.

Overall, 109 of 2873 patients (3.8%) were identified with a CHD and T21, with 49.5% patients being female. The mean age was 12.9 ± 4.4 years. Of these 109 T21 CHD patients, 21 patients (19.3%) had a simple CHD, 65 (59.6%) had a moderate CHD, and 23 patients (21.1%) had a severe CHD.

**Figure 1:**
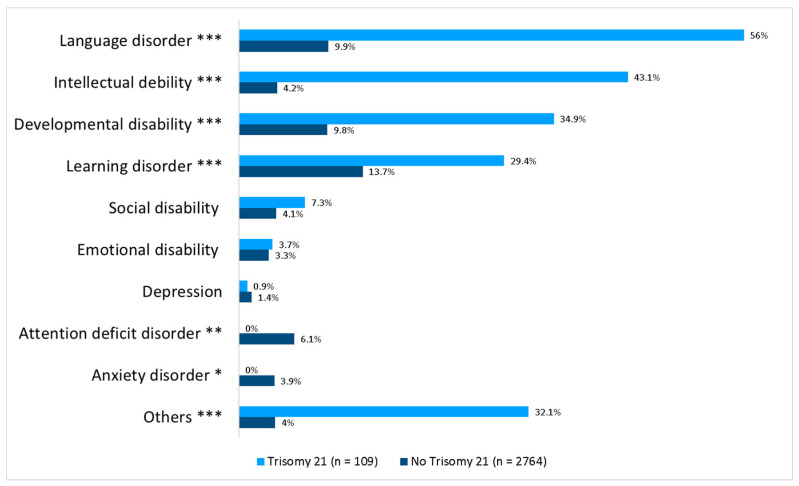
Psychological disorders in CHD patients with/without trisomy 21. *** denotes *p* < 0.001; ** denotes *p* < 0.01; * denotes *p* < 0.05.

A psychological disorder was reported in 33.3% of patients. T21 CHD patients had a psychological disorder significantly more often (82.6% vs. 31.4%, *p* < 0.001). Figure 1 gives a detailed overview of the reported psychological comorbidities.

### 3.2. Educational Status

#### Period before School Enrollment

**Figure 2:**
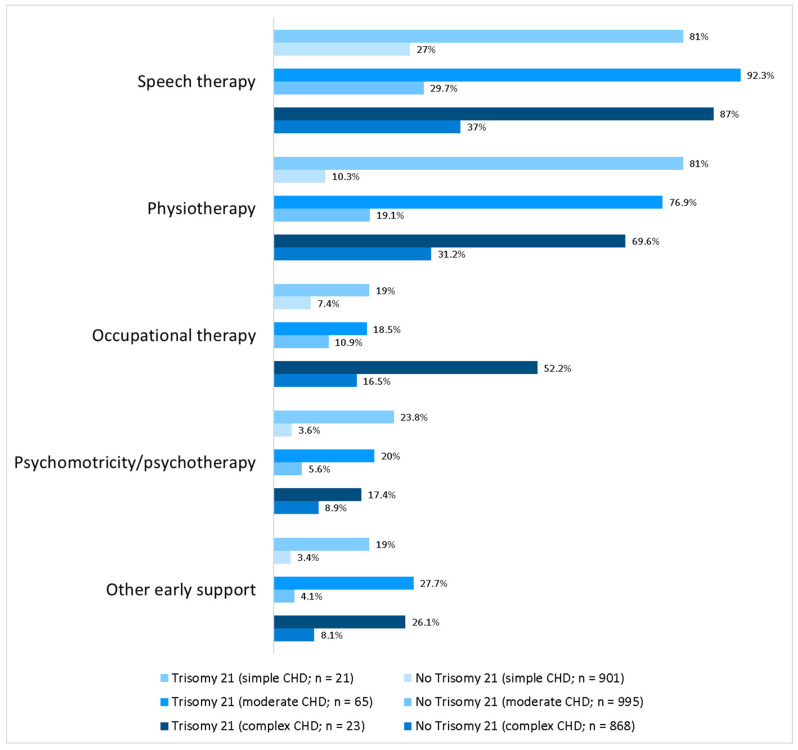
Support measures in CHD patients with and without trisomy 21; percentage of patients using support measures including division into different CHD severity groups; all differences between patients with and without trisomy 21 are significant (*p* < 0.001).

Half of the cohort (52%) of nT21 CHD patients (*n* = 2764) received early supportive measures (speech/occupational therapy, physio- or psychotherapy) in contrast to the T21 CHD cohort, where every patient (100%) received additive support even more frequently (≥3 early supportive measures 7.1% vs. 44%; *p* < 0.001). Figure 2 gives a detailed overview of administered supportive measures.

The estimated cumulative hospitalization time before school enrollment was significantly higher with increasing CHD severity in the overall cohort (*p* < 0.001): 85.4% with simple, 53.1% with moderate, and 18.4% with complex CHD reported a length of hospital stay < 1 month, while 0.5% with simple, 3.9% with moderate, and 17.5% with complex CHD reported >7 months. In T21 CHD patients, a tendency towards moderate, but not low, hospitalization time was observed. In total, 49.5% of the T21 CHD patients had a length of hospital stay <1 month compared to 52.8% of the nT21 CHD patients, and 47.7% of the T21 CHD patients had clinic time before school enrollment of 1–7 months compared to 36% of the nT21 CHD patients (*p* < 0.05).

### 3.3. School Career

**Figure 3:**
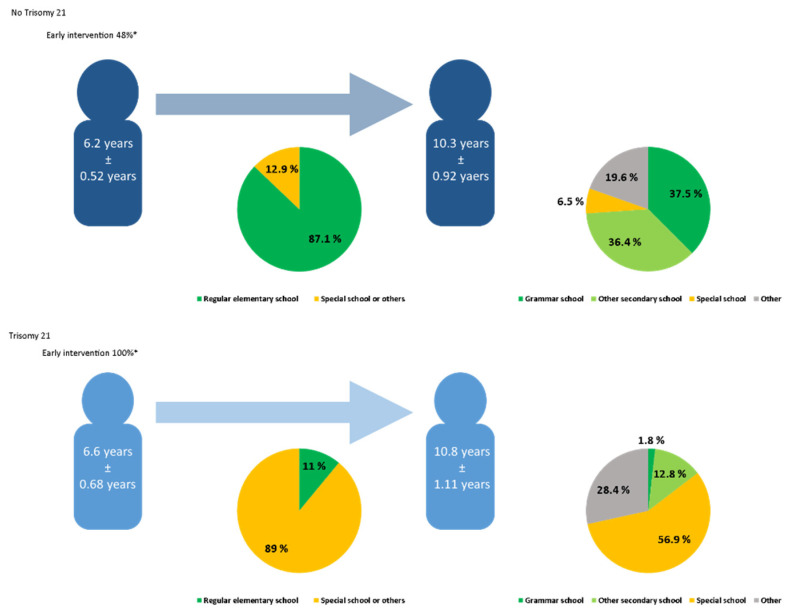
School enrollment and school transfer to secondary school; * Significant differences in early intervention between patients with and without trisomy 21 (*p* < 0.001); In the last pie chart, the added percentages add up to 99.9% and not 100%, as only one decimal place is given and therefore inaccuracies arise due to rounding up or down.

The educational career of and usage of supportive interventions for nT21CHD patients compared to T21 CHD patients is visualized in Figure 3. The school enrollment for both groups was similar at approximately 6 years of age. However, the T21 CHD patients tended to be enrolled later on average than the nT21 CHD patients (*p* < 0.001). Overall, 87.1% of the nT21 CHD patients but 11% of the T21 CHD patients were enrolled in a regular elementary school (*p* < 0.001). Also, 32.1% of the T21 CHD patients were enrolled in an integration class (2.9% in nT21 CHD patients). About 4 years later, at the age of 10 years, children of both groups went to a secondary school, with T21 CHD patients being slightly older (*p* < 0.01): 37.5% of the nT21 CHD patients went to grammar school and 6.5% needed specialized schools for special needs. In contrast, only 1.8% of the T21 CHD patients went to a grammar school, while the proportion of pupils at special schools was more than eight-times higher than in the nT21 CHD patients (*p* < 0.001). Just 12.8% of the T21 CHD patients visited a secondary school.

In total, 78% of the T21 CHD patients and 28.4% of the nT21 CHD patients reported having ever received special education services (e.g., because of specific learning disabilities) for at least 3 months since entering school (*p* < 0.001). Being held back a year was statistically insignificant between the T21 CHD and nT21 CHD patients.

As shown in Table 3, in the overall cohort, with increasing CHD severity, children were less often enrolled at elementary schools and grammar schools, but more often at special needs schools, required supportive measures more frequently, and had longer absenteeism periods from school (*p* < 0.001).

### 3.4. Influence of Parental Educational Level

Besides somatic factors such as CHD severity and underlying syndromic condition, the parental educational level may influence the child’s educational pathway. In the overall cohort, one-third of patients each had a low, medium, or high parental education level. Parental educational level was not significantly different in children based on CHD severity (see Table 4).

The same holds true for the presence or absence of trisomy 21, However, there was a tendency (*p* = 0.063) for higher parental education in the T21 CHD patients (23.9% with low, 32.1% with moderate, and 44% with high parental education) compared with the nT21 CHD patients (33.1% with low, 32.5% moderate, and 34.4% high parental education). An overview is given in Table 3.

The utilization of early support measures was similar irrespective of the parental educational level (Table 5). Parental educational level was significantly associated with the utilization of regular schools and inclusive schooling (*p* < 0.001). The percentage of young CHD patients attending grammar school was higher with increasing parental educational level (low/moderate/high parental educational level = 25.5%/36.2%/46.1%; *p* < 0.001). Further details are presented in Table 5.

## 4. Discussion

To our knowledge, this analysis provides the first robust overview on schooling and education in T21 CHD patients and nT21 CHD patients. It is well proven in the scientific world that patients with CHD are at risk for impaired neurodevelopment and that this may affect their school education [8,9,10,11,12].

As our results show, on average, patients with a CHD perform satisfactorily in their school development. However, there are specific patient cohorts who are at risk for impaired school development [26]. A severe CHD and/or a genetic syndrome such as T21 seem to be aggravating factors. T21 goes along with heterogeneous organic and neurodevelopmental comorbidities, and the scholastic and occupational careers o T21 individuals are diverse [27].

Education is a particularly important and objectively measurable characteristic of neurodevelopment. In our study, T21 CHD participants had a high demand for early and specific interventions. The majority of T21 CHD patients were enrolled at a special school and, when transferring to a secondary school, they again mainly attended a special school. In comparison, according to the Federal Statistical Office, 93.6% of children in Germany started their school career at an elementary school in the 2022/2023 school year, while 3.2% started at special schools and 2.4% at other types of school [28].

However, a small part of the T21 CHD patients attended a secondary school or grammar school. The question is whether this small proportion of school-ready T21 CHD patients can be further increased with appropriately close supervision and support, both before school enrollment and during and outside of school, to eventually provide more T21 CHD children with greater independence and self-determination through education. However, this question can only be conclusively answered in future, prospective, randomized controlled, longitudinal studies.

To understand the etiology of our results, it is important to know the common pattern of neurodevelopment in children with T21. Generally, children with T21 have a cognitive function in the mild to moderate intellectual impairment range, with a mean IQ score of 50, and have a characteristic profile of speech and language deficits with impairments in both receptive and expressive language [29,30]. Regarding the school performance, T21 patients often have significant difficulties in reading and mathematics and require special assistance to improve their learning skills and academic achievement [18]. Additionally, this patient cohort often develops behavioral and emotional problems, including attention-deficit/hyperactivity disorder, and internalizing and externalizing symptoms [31]. These symptoms often even manifest in psychiatric disorders. However, the mental health of children and adult patients with T21 unfortunately often receives little attention in the scientific world, as shown by the fact that a large review paper mentions the topic only in passing [32]. The importance of paying more attention to this aspect is shown by data from a comprehensive analysis of psychiatric disorders in 6.078 T21 patients (age 0–89 years). The authors found elevated prevalence for the diagnosis categories anxiety disorders, obsessive compulsive disorders, affective disorders (especially unipolar depression), any psychotic disorders, schizophrenia, tic disorders, impulse control disorders, and dementia disorders [33]. However, there are also, albeit a small proportion of, T21 patients who, due to maximum (early) supportive measures, intrinsic functional capacities, and appropriate socio-economic background, have only a mild cognitive impairment and are able to graduate from school and participate in the workforce. This complex pattern of neurodevelopment in T21 CHD patients is affirmed by the present study results.

The main reason for school problems and cognition difficulties In patients with CHD in general and in T21 CHD patients might be the high prevalence of psychiatric disorders such as learning, emotional, or behavioral disorders, which are clustered in T21 CHD patients, whereas the severity of CHD does not have such massive consequences. Accordingly, for patients with CHD as well as for T21 CHD patients, a holistic treatment approach should be pursued in principle, which, in addition to purely medical care, also takes into account the psychological and social aspects and provides low-threshold counseling and therapeutic support services as needed. It is plausible that a reduction in mental illness and prevention of the manifestation of psychopathological diseases would lead to better school and educational results.

### Limitations

Due to the data privacy policy of the National Register for Congenital Heart Defects, a non-responder analysis could not be performed. Our results need to be interpreted in light of a potential selection bias. Highly educated and/or healthier CHD patients might be more inclined to participate in scientific studies than patients with lower educational levels and/or more health problems. However, the proportion of 31.0% of patients with a severe CHD refutes this assumption. In addition, pseudonymized participation counteracts the effect of socially desirable answers and can possibly also have a positive effect on the willingness to participate of more educationally disadvantaged groups. The results found cannot be easily transferred to other countries as education and health care systems may differ. Due to the study setting, we could not assess the highest educational/academic achievement as the mean age of our study cohort was ~14 years of age. Since the present study is a data analysis based on two online patient surveys (explorative cross-sectional studies), correlations can be reported, but not causal relationships. In addition, the results reflect the subjective statements of the respondents, but whether the information on school careers actually corresponds to reality can only be assumed, but not conclusively verified.

## 5. Conclusions

Patients with CHD are at risk for impaired educational development, with T21 being an aggravating factor. Routine follow-up examinations should be established to identify neurodevelopmental deficits. Unfortunately, underlying genetic variables are hardly modifiable. However, supportive therapy might be in some cases a promising compensation mechanism. Therefore, CHD patients and their families should be given low-threshold access to supportive interventions. Further studies are necessary to evaluate the impact of these interventions and to carry out long-term follow up this specific patient cohort at risk.

## Figures and Tables

**Table 1:** Sample characteristics.

	Total	Simple CHD	Moderate CHD	Complex CHD
	Male	Male	Male	Male
	Female	Female	Female	Female
Sample composition	2873 (100%)	922 (32.1%)	1060 (36.9%)	891 (31%)
	1423 (49.5%)	373 (40.5%)	513 (48.4%)	537 (60.3%)
	1450 (50.5%)	549 (59.5%)	547 (51.6%)	354 (39.7%)
No trisomy 21	2764 (96.2%)	901 (97.7%)	995 (93.9%)	868 (97.4%)
	1368 (49.5%)	359 (96.2%)	485 (94.5%)	524 (97.6%)
	1396 (50.5%)	542 (98.7%)	510 (93.2%)	344 (97.2%)
With trisomy 21	109 (3.8%)	21 (2.3%)	65 (6.1%)	23 (2.6%)
	55 (50.5%)	14 (3.8%)	28 (5.5%)	13 (2.4%)
	54 (49.5%)	7 (1.3%)	37 (6.8%)	10 (2.8%)
Average age	14.1 ± 4.7 years	13.1 ± 4 years	14.6 ± 4.9 years	14.3 ± 5 years
	13.9 ± 4.7 years	12.8 ± 3.9 years	14.3 ± 4.8 years	14.2 ± 4.9 years
	14.2 ± 4.8 years	13.4 ± 4.1 years	14.9 ± 5 years	14.5 ± 5 years

CHD = congenital heart defect; blue letters/numbers represent male patients and purple letters/numbers represent female patients.

**Table 2:** Overview of the most frequent CHD in the overall cohort.

Congenital Heart Defect (CHD)	*n* (%)
Ventricular septal defect (VSD)	715 (24.9%)
Atrial septal defect (ASD)	312 (10.9%)
Tetralogy of Fallot (TOF)	290 (10.1%)
Univentricular heart (UVH)	280 (9.7%)
Transposition of the great arteries (TGA)	238 (8.3%)
Coarctation of the aorta (CoA)	201 (7%)
Aortic valve disease, e.g., aortic valve stenosis (AoV)	183 (6.4%)
Atrioventricular septal defect (AVSD)	136 (4.7%)
Other CHD	518 (17.9%)

**Table 3:** Data after school enrollment (patient reports).

		Total	Simple CHD	Moderate CHD	Complex CHD	No Trisomy 21	With Trisomy 21
		Male	Male	Male	Male	Male	Male
		Female	Female	Female	Female	Female	Female
		*N* = 2873	***N* = 922**	***N* = 1060**	***N* = 891**	***N* = 2764**	***N* = 109**
		*n* = 1423	*n* = 373	*n* = 513	*n* = 537	*n* = 1368	*n* = 55
		*n* = 1450	*n* = 549	*n* =547	*n* = 354	*n* = 1396	*n* = 54
School enrollment (average age in years) ^1^		6.2 ± 0.53	**6.1 ± 0.49** ^**sm**^^**&****sc**^	**6.2 ± 0.55** ^**mc**^	**6.3 ± 0.54**	**6.2 ± 0.52** ^**NTWT**^	**6.6 ± 0.68**
		**6.3 ± 0.55**	6.1 ± 0.5	**6.3 ± 0.56**	**6.3 ± 0.55**	**6.2 ± 0.53**	6.7 ± 0.72
		**6.1 ± 0.52**	6.1 ± 0.49	**6.2 ± 0.54**	**6.2 ± 0.51**	**6.1 ± 0.5**	6.6 ± 0.64
School type to which enrollment occurred ^2^	Elementary school	84.2%	**92.1%** ^**smc**^	**83%**	**77.5%**	**87.1%** ^**NTWT**^	**11%**
		**81.4%**	90.9%	82%	74.9%	**84.5%**	12.7%
		**86.4%**	92.9%	83.8%	81.3%	**89.7%**	9.3%
	Elementary school (ic)	4%	**2.2%**	**4.9%**	**4.7%**	**2.9%**	**32.1%**
		**4.1%**	2.4%	4.3%	5.2%	**3%**	32.7%
		3.8%	2%	5.5%	4%	**2.7%**	31.5%
	Special school	8.4%	**3.7%**	**8.1%**	**13.8%**	**7%**	**44%**
		**10.4%**	5.4%	9%	15.4%	**9.1%**	43.6%
		6.5%	2.6%	7.4%	11.3%	**5%**	44.4%
	Alternative school	3.4%	**2.1%**	**4%**	**4.1%**	**3%**	**12.8%**
		**3.7%**	1.3%	4.7%	4.5%	**3.4%**	10.9%
		**3%**	2.6%	3.3%	3.4%	**2.6%**	14.8%
Transfer to secondary school	Still visit primary school ^a^	15.1%	**16.2%** ^**smc**^	**13.8%**	**15.6%**	**15.2%** ^**NTWT**^	**13.8%**
		**15.9%**	15.5%	16.2%	14.7%	**15.9%**	16.4%
		**14.3%**	17.2%	11.5%	16.9%	**14.5%**	11.1%
	Special school (c/s)	8.4%	**3%**	**8.9%**	**13.4%**	**6.5%**	**56.9%**
		**9.9%**	3.8%	9.2%	14.9%	**8%**	56.4%
		**6.9%**	2.6%	8.6%	11%	**4.9%**	57.4%
	Alternative school (c/s)	2.3%	**1.2%**	**2.6%**	**2.9%**	**1.8%**	**12.8%**
		**2.5%**	0.8%	2.7%	3.5%	**2.1%**	12.7%
		**2%**	1.5%	2.6%	2%	**1.6%**	13%
	Secondary school	35.5%	**33.6%**	**34.8%**	**38.3%**	**36.4%**	**12.8**%
		**34.6%**	34.9%	32%	36.9%	**35.5%**	10.9%
		**36.4%**	32.8%	37.5%	40.4%	**37.2%**	14.8%
	Grammar school	36.1%	**43.7%**	**36.9%**	**27.4%**	**37.5%**	**1.8%**
		**34.5%**	41.6%	36.6%	27.6%	**35.8%**	1.8%
		**37.7%**	45.2%	37.1%	27.1%	**39.1%**	1.9%
	Other/do not know	2.6%	**2.3%**	**3%**	**2.5%**	**2.6%**	**1.8%**
		**2.6%**	1.9%	3.3%	2.4%	**2.6%**	1.8%
		**2.6%**	2.6%	2.7%	2.5%	**2.7%**	1.9%
School transfer (average age in years) ^3^		10.3 ± 0.9	**10.2 ± 0.7** ^**sm****&****sc**^	**10.3 ± 0.9** ^**mc**^	**10.5 ± 1.1**	**10.3 ± 0.9** ^**NTWT**^	**10.8 ± 1.1**
		10.4 ± 0.9	10.3 ± 0.7	10.3 ± 0.8	10.5 ± 1.1	10.4 ± 0.9	10.6 ± 0.8
		10.3 ± 0.9	10.2 ± 0.7	10.3 ± 0.96	10.4 ± 1.2	10.3 ± 0.9	11 ± 1.3
A class had to be repeated at least once		10.9%	**7.3%** ^**smc**^	**10.7%**	**15%**	11%	10.1%
		**12.2%**	7.8%	10.5%	16.8%	12.4%	5.5%
		**9.7%**	6.9%	10.8%	12.4%	**9.5%**	14.8%
Absenteeism from school due to CHD	<1 month	79.2%	**90%** ^**smc**^	**80.8%**	**65.9%**	**78.7%** ^**NTWT**^	**90.8%**
		**78.1%**	91.7%	81.7%	65.4%	**77.7%**	89.1%
		**80.1%**	88.9%	80.1%	66.6%	**79.7%**	92.6%
	1 to 3 months	9.3%	**2.6%**	**9.6%**	**15.9%**	**9.6%**	**3.7%**
		**10.5%**	2.4%	9.9%	16.6%	**10.7%**	3.6%
		**8.2%**	2.7%	9.3%	15%	**8.4%**	3.7%
	≥4 months	5.7%	**0.8%**	**3.8%**	**13%**	**5.9%**	-
		**6.5%**	0.8%	3.9%	13%	**6.8%**	
		**4.8%**	0.7%	3.7%	13%	**5%**	
	Do not know	5.8%	**6.6%**	**5.8%**	**5.2%**	**5.9%**	**5.5%**
		**4.8%**	5.1%	4.5%	5%	**4.8%**	7.3%
		**6.8%**	7.7%	6.9%	5.4%	**6.9%**	3.7%
≥3 months support since attending school		30.2%	**23.2%** ^**smc**^	**30.4%**	**37.4%**	**28.4%** ^**NTWT**^	**78%**
		**34%**	27.1%	32.9%	39.9%	**32.3%**	76.4%
		**26.6%**	20.6%	28%	33.6%	**24.5%**	79.6%

CHD = congenital heart defect; c/s = change from primary school to secondary school or stay at primary school without school change; ic = integration class; ^1^ reduced sample size because 45 patients did not know their enrollment age at the time of data collection (total = 2828/female = 1428; simple CHD = 918/female = 546; moderate CHD = 1042/female = 536; complex CHD = 868/female = 346; no trisomy 21 = 2722/female = 1375; with trisomy 21 = 106/female = 53); ^2^ reduced sample size because 8 patients did not know their type of school at enrollment at the time of data collection (total = 2865/female = 1446; simple CHD = 922/female = 549; moderate CHD = 1056/female = 544; complex CHD = 887/female = 353; no trisomy 21 = 2756/female = 1392; with trisomy 21 = 109/female = 54); ^a^ no change so far, still regularly visit primary school (elementary school/elementary school ic); ^3^ reduced sample size because age at school transfer was not true/known/stated for all patients (total = 2176/female = 1131; simple CHD = 733/female = 443; moderate CHD = 810/female = 433; complex CHD = 633/female = 255; no trisomy 21 = 2138/female = 1110; with trisomy 21 = 38/female = 21); significant sex differences within a group: *p* < 0.001/*p* < 0.01/*p* < 0.05; ^smc/sc/mc/NTWT^ significant differences between simple, moderate, and complex CHD (smc), simple and complex CHD (sc), moderate and complex CHD (mc) in patients without and with trisomy 21 (NTWT) colored in green (*p* < 0.001), orange (*p* < 0.01), and red (*p* < 0.05), measured using *t*-test or χ^2^-test; numbers in bold indicate significant group differences or gender differences within groups; blue letters/numbers represent male patients and purple letters/numbers represent female patients.

**Table 4:** Parents’ educational level based on ISCED taking into account CHD severity and trisomy 21.

	Total	Simple CHD	Moderate CHD	Complex CHD	No Trisomy 21	With Trisomy 21
	Male	Male	Male	Male	Male	Male
	Female	Female	Female	Female	Female	Female
	***N* = 2873**	***N* = 922**	***N* = 1060**	***N* = 891**	***N* = 2764**	***N* = 109**
	*n* = 1423	*n* = 373	*n* = 513	*n* = 537	*n* = 1368	*n* = 55
	*n* = 1450	*n* = 549	*n* =547	*n* = 354	*n* = 1396	*n* = 54
Low education	941 (32.8%)	285 (30.9%)	354 (33.4%)	302 (33.9%)	915 (33.1%)	26 (23.9%)
	446 (31.3%)	104 (27.9%)	161 (31.4%)	181 (33.7%)	433 (31.7%)	13 (23.6%)
	495 (34.1%)	181 (33%)	193 (35.3%)	121 (34.2%)	482 (34.5%)	13 (24.1%)
Moderate education	932 (32.4%)	317 (34.4%)	339 (31.5%)	281 (31.5%)	897 (32.5%)	35 (32.1%)
	446 (31.3%)	124 (33.2%)	157 (30.6%)	165 (30.7%)	426 (31.1%)	20 (36.4%)
	486 (33.5%)	193 (35.2%)	177 (32.4%)	116 (32.8%)	471 (33.7%)	15 (27.8%)
High education	1000 (34.8%)	320 (34.7%)	372 (35.1%)	308 (34.6%)	952 (34.4%)	48 (44%)
	531 (37.3%)	145 (38.9%)	195 (38%)	191 (35.6%)	509 (37.2%)	22 (40%)
	469 (32.3%)	175 (31.9%)	177 (32.4%)	117 (33.1%)	443 (31.7%)	26 (48.1%)

CHD = congenital heart defect; parental education groups did not differ significantly with respect to CHD severity (*p* = 0.547); parental education groups did not differ significantly with respect to patients with trisomy 21/no trisomy 21 (*p* = 0.063); blue letters/numbers represent male patients and purple letters/numbers represent female patients.

**Table 5:** Parents’ educational level based on ISCED against the background of the child’s educational pathway.

		Low Parental Education Level (*n* = 941)	Moderate Parental Education Level (*n* = 932)	High Parental Education Level (*n* = 1000)	*p*-Value
CHD prenatally diagnosed		149 (15.8%)	153 (16.4%)	204 (20.4%)	*p* < 0.05
Early support measures before school enrollment		457 (48.6%)	484 (51.9%)	494 (49.4%)	-
School enrollment (average age in years) ^1^		6.23 ± 0.536	6.21 ± 0.522	6.15 ± 0.539	low vs. high (*p* < 0.01) moderate vs. high (*p* < 0.01)
Primary school (type of school at enrollment) ^2^	Elementary school	792 (84.5%)	774 (83.1%)	846 (84.9%)	*p* < 0.001
	Elementary school (ic)	26 (2.8%)	40 (4.3%)	48 (4.8%)	
	Special school	105 (11.2%)	86 (9.2%)	51 (5.1%)	
	Alternative school	14 (1.5%)	31 (3.3%)	52 (5.2%)	
Transfer to secondary school	Still visit primary school ^a^	137 (14.6%)	131 (14.1%)	166 (16.6%)	*p* < 0.001
	Special school (c/s)	92 (9.8%)	90 (9.7%)	59 (5.9%)	
	Alternative school (c/s)	12 (1.3%)	18 (1.9%)	35 (3.5%)	
	Secondary school	431 (45.8%)	339 (36.4%)	250 (25%)	
	Grammar school	240 (25.5%)	337 (36.2%)	461 (46.1%)	
	Other/do not know	29 (3.1)	17 (1.8%)	29 (2.9%)	
School transfer (average age in years) ^3^		10.34 ± 0.97 years	10.33 ± 0.92 years	10.27 ± 0.88 years	low vs. high (*p* < 0.05)
A class had to be repeated at least once		132 (14%)	89 (9.5%)	93 (9.3%)	*p* < 0.01
≥3 months support since attending school		286 (30.4%)	296 (31.8%)	287 (28.7%)	-

CHD = congenital heart defect; c/s = change from primary school to secondary school or stay at primary school without school change; ic = integration class; ^1^ reduced sample size because 45 patients did not know their enrollment age at the time of data collection (total = 2828/female = 1428; simple CHD = 918/female = 546; moderate CHD = 1042/female = 536; complex CHD = 868/female = 346; no trisomy 21 = 2722/female = 1375; with trisomy 21 = 106/female = 53); ^2^ reduced sample size because 8 patients did not know their type of school at enrollment at the time of data collection (total = 2865/female = 1446; simple CHD = 922/female = 549; moderate CHD = 1056/female = 544; complex CHD = 887/female = 353; no trisomy 21 = 2756/female = 1392; with trisomy 21 = 109/female = 54); ^a^ no change so far, still regularly visit primary school (elementary school/elementary school ic); ^3^ reduced sample size because age at school transfer was not true/known/stated for all patients (total = 2176/female = 1131; simple CHD = 733/female = 443; moderate CHD = 810/female = 433; complex CHD = 633/female = 255; no trisomy 21 = 2138/female = 1110; with trisomy 21 = 38/female = 21); significant group differences colored in green (*p* < 0.001), orange (*p* < 0.01), and red (*p* < 0.05), measured using *t*-test or χ^2^-test.

## Data Availability

Data are contained within the article.
